# {μ-6,6′-Dimeth­oxy-2,2′-[propane-1,3-diylbis(nitrilo­methyl­idyne)]diphenolato}dimethanoltrinitratonickel(II)praseodymium(III) methanol disolvate

**DOI:** 10.1107/S1600536808005357

**Published:** 2008-03-29

**Authors:** Fei Liu, Fang Zhang

**Affiliations:** a The College of Chemical Engineering & Materials, Eastern Liaoning University, 325 Wenhua Road, Yuanbao District, Dandong City, Liaoning Province 118003, People’s Republic of China

## Abstract

In the title dinuclear complex, [NiPr(C_19_H_20_N_2_O_4_)(NO_3_)_3_(CH_3_OH)_2_]·2CH_3_OH, the Ni^II^ ion is coordinated by two O atoms and two N atoms of a Schiff base ligand and by two methanol ligands, forming a slightly distorted octa­hedral geometry. The Pr^III^ ion is coordinated by six O atoms from three chelating nitrate ligands and four O atoms from a Schiff base ligand, forming a distorted bicapped square-anti­prismatic environment. In the crystal structure, inter­molecular O—H⋯O hydrogen bonds connect complex mol­ecules and methanol solvent mol­ecules to form (10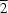) sheets.

## Related literature

For related crystal structures, see: Elmali & Elerman (2003 ▶, 2004 ▶).
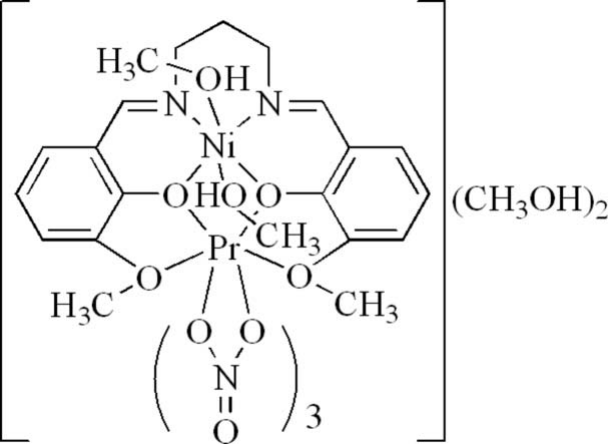


## Experimental

### 

#### Crystal data

[NiPr(C_19_H_20_N_2_O_4_)(NO_3_)_3_(CH_4_O)_2_]·2CH_4_O
*M* *_r_* = 854.17
Monoclinic, 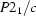

*a* = 13.101 (3) Å
*b* = 11.128 (2) Å
*c* = 22.213 (4) Å
β = 90.73 (3)°
*V* = 3238.1 (11) Å^3^
*Z* = 4
Mo *K*α radiation
μ = 2.15 mm^−1^
*T* = 293 (2) K
0.33 × 0.31 × 0.20 mm

#### Data collection

Rigaku R-AXIS RAPID diffractometer
Absorption correction: multi-scan (*ABSCOR*; Higashi, 1995 ▶) *T*_min_ = 0.536, *T*_max_ = 0.674
30138 measured reflections
7364 independent reflections
6223 reflections with *I* > 2σ(*I*)
*R*_int_ = 0.036

#### Refinement

*R*[*F* ^2^ > 2σ(*F* ^2^)] = 0.029
*wR*(*F* ^2^) = 0.067
*S* = 1.03
7364 reflections
430 parameters
H-atom parameters constrained
Δρ_max_ = 0.68 e Å^−3^
Δρ_min_ = −0.34 e Å^−3^

### 

Data collection: *RAPID-AUTO* (Rigaku, 1998 ▶); cell refinement: *RAPID-AUTO* data reduction: *CrystalStructure* (Rigaku/MSC, 2002 ▶); program(s) used to solve structure: *SHELXS97* (Sheldrick, 2008 ▶); program(s) used to refine structure: *SHELXL97* (Sheldrick, 2008 ▶); molecular graphics: *SHELXTL* (Sheldrick, 2008 ▶); software used to prepare material for publication: *SHELXL97*.

## Supplementary Material

Crystal structure: contains datablocks global, I. DOI: 10.1107/S1600536808005357/lh2586sup1.cif

Structure factors: contains datablocks I. DOI: 10.1107/S1600536808005357/lh2586Isup2.hkl

Additional supplementary materials:  crystallographic information; 3D view; checkCIF report

## Figures and Tables

**Table 1:** Hydrogen-bond geometry (Å, °)

*D*—H⋯*A*	*D*—H	H⋯*A*	*D*⋯*A*	*D*—H⋯*A*
O14—H14*O*⋯O16	0.85	1.81	2.661 (4)	180
O15—H15*O*⋯O6^i^	0.85	2.28	3.128 (5)	180
O16—H16*O*⋯O17^ii^	0.85	1.87	2.720 (6)	179
O17—H17*O*⋯O13	0.85	2.05	2.905 (5)	180

Symmetry codes: (i) 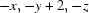; (ii) 
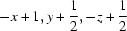
.

**Fig. 1.**
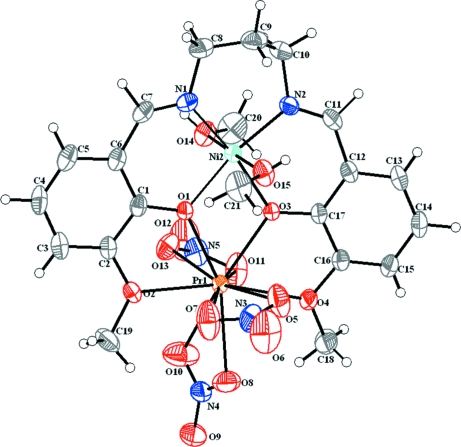
The molecular structure of the title compound showing 40% probability displacement ellipsoids. The solvent methanol molecules have been omitted for clarity.

## supplementary crystallographic information

### Comment

The molecular structure is shown in Fig. 1. The hexadentate Schiff base ligand links Ni^II^ and Pr^III^ ions to form a dinuclear complex *via* two bridging phenolate O atoms, similar to reported copper-lanthanum complexes of the same ligand (Elmali & Elerman (2003,2004). The Pr^III^ ion is ten-coordinated by four O atoms from the Schiff base ligand and six O atoms from three chelating nitrate ligands. The Ni^II^ ion is coordinated by two N atoms and two O atoms from the Schiff base ligand and two methanol oxygen atoms. In the crystal structure, intermolecular O—H···O hydrogen bonds connect complex molecules and methanol solvent molecules to form (1 0 - 2) sheets.

### Experimental

The title complex was obtained by the treatment of Ni(II)acetate tetrahydrate (0.0622 g,0.25 mmol) with the Schiff base (0.0855 g,0.25 mmol) in methanol (25 ml) at room temperature. Then the mixture was refluxed for 3 h after the addition of praseodymium (III) nitrate hexahydrate (0.1042 g, 0.25 mmol). The reaction mixture was cooled and filtered; diethyl ether was allowed to diffuse slowly into the solution of the filtrate. Single crystals were obtained after several days. Analysis calculated for C_23_H_34_NiN_5_O_17_Pr: C, 32.38; H, 4.12; N, 8.18; found: C, 32.42; H, 4.02; N, 8.22

### Refinement

H atoms bound to C atoms were placed in calculated positions and treated as riding on their parent atoms, with C—H = 0.93 Å (aromatic C), C—H = 0.97 Å (methylene C), C—H = 0.98 Å (methine C), and with *U*_iso_(H) = 1.2*U*_eq_(C) or C—H = 0.96 Å (methyl C) and with *U*_iso_(H) = 1.5*U*_eq_(C). H atoms bonded to O atoms were placed in calculated positions which gave the theoretically best locations to be involved in hydrogen bonding and treated as riding on their parent atoms, with O—H = 0.85 Å, and with *U*_iso_(H) = 1.5*U*_eq_(O).

### Crystal data

**Table:**

[NiPr(C_19_H_20_N_2_O_4_)(NO_3_)_3_(CH_4_O)_2_]·2CH_4_O	*F*_000_ = 1728
*M**_r_* = 854.17	*D*_x_ = 1.752 Mg m^−^^3^
Monoclinic, *P*2_1_/*c*	Mo *K*α radiation λ = 0.71073 Å
Hall symbol: -P 2ybc	Cell parameters from 24596 reflections
*a* = 13.101 (3) Å	θ = 3.0–27.5º
*b* = 11.128 (2) Å	µ = 2.15 mm^−^^1^
*c* = 22.213 (4) Å	*T* = 293 (2) K
β = 90.73 (3)º	Block, green
*V* = 3238.1 (11) Å^3^	0.33 × 0.31 × 0.20 mm
*Z* = 4	

### Data collection

**Table:**

Rigaku R-AXIS RAPID diffractometer	7364 independent reflections
Radiation source: fine-focus sealed tube	6223 reflections with *I* > 2σ(*I*)
Monochromator: graphite	*R*_int_ = 0.036
*T* = 293(2) K	θ_max_ = 27.5º
ω scans	θ_min_ = 3.0º
Absorption correction: multi-scan(ABSCOR; Higashi, 1995)	*h* = −17→16
*T*_min_ = 0.536, *T*_max_ = 0.674	*k* = −14→12
30138 measured reflections	*l* = −28→28

### Refinement

**Table:**

Refinement on *F*^2^	Secondary atom site location: difference Fourier map
Least-squares matrix: full	Hydrogen site location: inferred from neighbouring sites
*R*[*F*^2^ > 2σ(*F*^2^)] = 0.029	H-atom parameters constrained
*wR*(*F*^2^) = 0.067	*w* = 1/[σ^2^(*F*_o_^2^) + (0.0247*P*)^2^ + 3.534*P*] where *P* = (*F*_o_^2^ + 2*F*_c_^2^)/3
*S* = 1.03	(Δ/σ)_max_ = 0.004
7364 reflections	Δρ_max_ = 0.68 e Å^−^^3^
430 parameters	Δρ_min_ = −0.34 e Å^−^^3^
Primary atom site location: structure-invariant direct methods	Extinction correction: none

### Special details

**Table:**

Geometry. All e.s.d.'s (except the e.s.d. in the dihedral angle between two l.s. planes) are estimated using the full covariance matrix. The cell e.s.d.'s are taken into account individually in the estimation of e.s.d.'s in distances, angles and torsion angles; correlations between e.s.d.'s in cell parameters are only used when they are defined by crystal symmetry. An approximate (isotropic) treatment of cell e.s.d.'s is used for estimating e.s.d.'s involving l.s. planes.
Refinement. Refinement of *F*^2^ against ALL reflections. The weighted *R*-factor *wR* and goodness of fit *S* are based on *F*^2^, conventional *R*-factors *R* are based on *F*, with *F* set to zero for negative *F*^2^. The threshold expression of *F*^2^ > σ(*F*^2^) is used only for calculating *R*-factors(gt) *etc*. and is not relevant to the choice of reflections for refinement. *R*-factors based on *F*^2^ are statistically about twice as large as those based on *F*, and *R*- factors based on ALL data will be even larger.

### Fractional atomic coordinates and isotropic or equivalent isotropic displacement parameters (Å^2^)

**Table:**

	*x*	*y*	*z*	*U*_iso_*/*U*_eq_	
Pr1	0.216569 (12)	0.702799 (13)	0.077562 (7)	0.03122 (5)	
Ni2	0.17232 (3)	0.98440 (3)	0.146000 (15)	0.03016 (8)	
O1	0.28024 (14)	0.90073 (17)	0.09639 (9)	0.0331 (4)	
O2	0.40132 (16)	0.7479 (2)	0.04462 (10)	0.0443 (5)	
O3	0.10030 (14)	0.82365 (17)	0.13252 (9)	0.0327 (4)	
O4	0.03400 (16)	0.61709 (18)	0.09151 (10)	0.0412 (5)	
O5	0.0847 (2)	0.7706 (3)	−0.00279 (12)	0.0713 (8)	
O6	0.1076 (3)	0.8251 (4)	−0.09346 (14)	0.1064 (13)	
O7	0.2344 (2)	0.7767 (3)	−0.03470 (14)	0.0868 (10)	
O8	0.2129 (3)	0.5235 (3)	0.00588 (16)	0.0864 (11)	
O9	0.2963 (3)	0.3558 (3)	0.01513 (19)	0.0981 (12)	
O10	0.3143 (3)	0.4978 (3)	0.07861 (15)	0.0910 (11)	
O11	0.1916 (2)	0.5814 (3)	0.17783 (13)	0.0689 (8)	
O12	0.3064 (3)	0.5697 (4)	0.24869 (14)	0.1008 (12)	
O13	0.3325 (2)	0.6765 (2)	0.16994 (11)	0.0538 (6)	
O14	0.23893 (18)	0.9184 (2)	0.22722 (9)	0.0477 (5)	
H14O	0.2809	0.9580	0.2489	0.072*	
O15	0.10034 (18)	1.0504 (2)	0.06495 (9)	0.0491 (6)	
H15O	0.0438	1.0841	0.0727	0.074*	
N1	0.2617 (2)	1.1332 (2)	0.15446 (11)	0.0388 (6)	
N2	0.0495 (2)	1.0458 (2)	0.19041 (10)	0.0366 (5)	
N3	0.1414 (3)	0.7903 (3)	−0.04484 (14)	0.0579 (8)	
N4	0.2736 (2)	0.4548 (3)	0.03316 (16)	0.0560 (8)	
N5	0.2774 (3)	0.6073 (3)	0.20013 (14)	0.0566 (8)	
C1	0.3769 (2)	0.9339 (3)	0.09110 (12)	0.0333 (6)	
C2	0.4459 (2)	0.8550 (3)	0.06371 (13)	0.0374 (6)	
C3	0.5472 (3)	0.8817 (3)	0.05670 (16)	0.0499 (8)	
H3A	0.5904	0.8274	0.0380	0.060*	
C4	0.5842 (3)	0.9906 (4)	0.07772 (18)	0.0567 (9)	
H4A	0.6530	1.0094	0.0737	0.068*	
C5	0.5199 (3)	1.0699 (3)	0.10423 (17)	0.0540 (9)	
H5A	0.5455	1.1428	0.1181	0.065*	
C6	0.4161 (2)	1.0446 (3)	0.11116 (13)	0.0395 (7)	
C7	0.3546 (3)	1.1377 (3)	0.13829 (14)	0.0413 (7)	
H7A	0.3873	1.2108	0.1448	0.050*	
C8	0.2245 (3)	1.2444 (3)	0.18456 (18)	0.0564 (9)	
H8A	0.2503	1.3137	0.1631	0.068*	
H8B	0.2525	1.2471	0.2252	0.068*	
C9	0.1106 (3)	1.2537 (3)	0.18762 (17)	0.0558 (9)	
H9A	0.0827	1.2504	0.1470	0.067*	
H9B	0.0932	1.3315	0.2043	0.067*	
C10	0.0603 (3)	1.1579 (3)	0.22453 (16)	0.0527 (9)	
H10A	0.1008	1.1432	0.2606	0.063*	
H10B	−0.0066	1.1855	0.2367	0.063*	
C11	−0.0395 (2)	1.0003 (3)	0.18771 (13)	0.0403 (7)	
H11A	−0.0911	1.0428	0.2067	0.048*	
C12	−0.0691 (2)	0.8885 (3)	0.15801 (12)	0.0355 (6)	
C13	−0.1738 (2)	0.8634 (3)	0.15589 (15)	0.0444 (7)	
H13A	−0.2194	0.9190	0.1714	0.053*	
C14	−0.2104 (2)	0.7590 (3)	0.13152 (16)	0.0501 (8)	
H14A	−0.2804	0.7448	0.1295	0.060*	
C15	−0.1421 (2)	0.6738 (3)	0.10978 (14)	0.0420 (7)	
H15A	−0.1661	0.6014	0.0941	0.050*	
C16	−0.0397 (2)	0.6973 (3)	0.11158 (12)	0.0341 (6)	
C17	0.0003 (2)	0.8059 (3)	0.13400 (12)	0.0312 (6)	
C18	0.0043 (3)	0.4945 (3)	0.0871 (2)	0.0624 (11)	
H18A	−0.0303	0.4712	0.1231	0.094*	
H18B	0.0639	0.4453	0.0822	0.094*	
H18C	−0.0406	0.4842	0.0530	0.094*	
C19	0.4576 (3)	0.6741 (3)	0.0038 (2)	0.0649 (11)	
H19A	0.4859	0.7234	−0.0273	0.097*	
H19B	0.4128	0.6153	−0.0140	0.097*	
H19C	0.5117	0.6340	0.0253	0.097*	
C20	0.1849 (4)	0.8569 (4)	0.27271 (18)	0.0739 (13)	
H20A	0.2319	0.8112	0.2970	0.111*	
H20B	0.1358	0.8038	0.2544	0.111*	
H20C	0.1503	0.9141	0.2976	0.111*	
C21	0.1500 (3)	1.0961 (4)	0.01370 (16)	0.0643 (11)	
H21A	0.1109	1.0764	−0.0218	0.096*	
H21B	0.2168	1.0611	0.0110	0.096*	
H21C	0.1561	1.1818	0.0171	0.096*	
O16	0.3699 (3)	1.0418 (3)	0.29578 (16)	0.1010 (12)	
H16O	0.4077	1.0979	0.2828	0.151*	
C22	0.4115 (5)	0.9988 (7)	0.3500 (3)	0.127 (2)	
H22A	0.4757	0.9605	0.3424	0.191*	
H22B	0.3655	0.9417	0.3674	0.191*	
H22C	0.4217	1.0646	0.3773	0.191*	
O17	0.5098 (3)	0.7213 (4)	0.2466 (2)	0.1223 (15)	
H17O	0.4579	0.7078	0.2242	0.183*	
C23	0.5316 (4)	0.8313 (5)	0.2238 (2)	0.0912 (15)	
H23A	0.4757	0.8849	0.2311	0.137*	
H23B	0.5422	0.8248	0.1813	0.137*	
H23C	0.5923	0.8620	0.2430	0.137*	

### Atomic displacement parameters (Å^2^)

**Table:**

	*U*^11^	*U*^22^	*U*^33^	*U*^12^	*U*^13^	*U*^23^
Pr1	0.02647 (8)	0.02928 (8)	0.03787 (9)	0.00184 (6)	−0.00064 (6)	−0.00402 (6)
Ni2	0.02980 (19)	0.02824 (17)	0.03245 (18)	0.00080 (14)	0.00038 (14)	−0.00305 (14)
O1	0.0260 (10)	0.0323 (10)	0.0412 (10)	−0.0017 (8)	0.0033 (8)	−0.0039 (8)
O2	0.0329 (12)	0.0451 (12)	0.0550 (13)	0.0028 (10)	0.0102 (10)	−0.0096 (10)
O3	0.0254 (10)	0.0331 (10)	0.0395 (10)	−0.0007 (8)	0.0023 (8)	−0.0058 (8)
O4	0.0332 (11)	0.0323 (11)	0.0581 (13)	−0.0039 (9)	0.0015 (10)	−0.0054 (9)
O5	0.0480 (16)	0.110 (2)	0.0565 (16)	0.0155 (16)	0.0019 (13)	0.0115 (16)
O6	0.097 (3)	0.156 (4)	0.066 (2)	0.010 (3)	−0.0267 (18)	0.047 (2)
O7	0.0560 (19)	0.133 (3)	0.0713 (19)	0.0041 (19)	−0.0019 (15)	0.0353 (19)
O8	0.074 (2)	0.073 (2)	0.112 (2)	0.0265 (17)	−0.0351 (19)	−0.0507 (18)
O9	0.069 (2)	0.0542 (18)	0.171 (3)	0.0109 (15)	0.006 (2)	−0.053 (2)
O10	0.130 (3)	0.0625 (19)	0.080 (2)	0.0355 (19)	−0.025 (2)	−0.0164 (16)
O11	0.0482 (16)	0.079 (2)	0.0792 (19)	−0.0052 (14)	−0.0038 (14)	0.0269 (15)
O12	0.090 (3)	0.141 (3)	0.071 (2)	0.005 (2)	−0.0150 (18)	0.055 (2)
O13	0.0548 (15)	0.0523 (14)	0.0540 (14)	−0.0093 (12)	−0.0132 (11)	0.0091 (11)
O14	0.0496 (14)	0.0531 (14)	0.0403 (12)	−0.0043 (11)	−0.0052 (10)	0.0072 (10)
O15	0.0446 (14)	0.0628 (15)	0.0399 (12)	0.0076 (11)	−0.0007 (10)	0.0083 (10)
N1	0.0467 (16)	0.0302 (13)	0.0394 (13)	−0.0045 (11)	−0.0014 (11)	−0.0027 (10)
N2	0.0371 (14)	0.0386 (13)	0.0342 (12)	0.0052 (11)	0.0007 (10)	−0.0062 (10)
N3	0.054 (2)	0.0603 (19)	0.0587 (19)	0.0014 (16)	−0.0162 (16)	0.0103 (15)
N4	0.0458 (18)	0.0390 (16)	0.084 (2)	−0.0042 (13)	0.0132 (16)	−0.0199 (15)
N5	0.056 (2)	0.0598 (19)	0.0539 (18)	0.0065 (16)	0.0000 (15)	0.0146 (15)
C1	0.0291 (15)	0.0411 (16)	0.0297 (13)	−0.0023 (12)	−0.0037 (11)	0.0061 (11)
C2	0.0286 (15)	0.0449 (17)	0.0387 (15)	−0.0006 (13)	0.0007 (12)	0.0042 (13)
C3	0.0310 (17)	0.061 (2)	0.058 (2)	0.0007 (15)	0.0055 (15)	0.0071 (17)
C4	0.0290 (17)	0.069 (2)	0.072 (2)	−0.0102 (17)	0.0045 (16)	0.0066 (19)
C5	0.043 (2)	0.055 (2)	0.064 (2)	−0.0178 (17)	−0.0035 (17)	0.0033 (17)
C6	0.0351 (17)	0.0419 (17)	0.0413 (16)	−0.0074 (13)	−0.0027 (13)	0.0049 (13)
C7	0.0466 (19)	0.0345 (16)	0.0427 (16)	−0.0128 (14)	−0.0052 (14)	0.0014 (12)
C8	0.064 (3)	0.0385 (18)	0.066 (2)	−0.0066 (17)	0.0016 (19)	−0.0156 (16)
C9	0.070 (3)	0.0344 (17)	0.062 (2)	0.0127 (17)	−0.0095 (19)	−0.0133 (16)
C10	0.052 (2)	0.050 (2)	0.056 (2)	0.0082 (16)	0.0050 (17)	−0.0218 (16)
C11	0.0379 (17)	0.0450 (17)	0.0380 (15)	0.0119 (14)	0.0065 (13)	−0.0019 (13)
C12	0.0302 (15)	0.0437 (16)	0.0326 (14)	0.0052 (13)	0.0039 (11)	0.0037 (12)
C13	0.0290 (16)	0.0530 (19)	0.0513 (18)	0.0068 (14)	0.0065 (13)	0.0035 (15)
C14	0.0255 (16)	0.068 (2)	0.057 (2)	−0.0038 (15)	0.0005 (14)	0.0039 (17)
C15	0.0322 (16)	0.0441 (17)	0.0496 (18)	−0.0067 (13)	−0.0009 (13)	−0.0017 (14)
C16	0.0305 (15)	0.0382 (15)	0.0336 (14)	0.0008 (12)	0.0004 (11)	0.0042 (12)
C17	0.0263 (13)	0.0379 (15)	0.0295 (13)	0.0010 (12)	0.0008 (10)	0.0045 (11)
C18	0.052 (2)	0.0362 (18)	0.100 (3)	−0.0080 (16)	0.009 (2)	−0.0035 (18)
C19	0.059 (2)	0.052 (2)	0.084 (3)	0.0058 (18)	0.032 (2)	−0.0128 (19)
C20	0.090 (3)	0.077 (3)	0.055 (2)	−0.014 (3)	0.002 (2)	0.019 (2)
C21	0.069 (3)	0.073 (3)	0.051 (2)	0.004 (2)	0.0041 (18)	0.0223 (19)
O16	0.107 (3)	0.101 (3)	0.093 (2)	−0.009 (2)	−0.046 (2)	−0.006 (2)
C22	0.128 (6)	0.160 (7)	0.092 (4)	0.022 (5)	−0.049 (4)	−0.010 (4)
O17	0.085 (3)	0.119 (3)	0.161 (4)	−0.011 (2)	−0.052 (3)	0.011 (3)
C23	0.085 (4)	0.096 (4)	0.093 (4)	0.014 (3)	−0.011 (3)	0.017 (3)

### Geometric parameters (Å, °)

**Table:**

Pr1—O3	2.3804 (19)	C4—H4A	0.9300
Pr1—O1	2.3903 (19)	C5—C6	1.399 (4)
Pr1—O8	2.552 (3)	C5—H5A	0.9300
Pr1—O13	2.554 (2)	C6—C7	1.448 (4)
Pr1—O5	2.580 (3)	C7—H7A	0.9300
Pr1—O2	2.587 (2)	C8—C9	1.498 (5)
Pr1—O4	2.597 (2)	C8—H8A	0.9700
Pr1—O10	2.616 (3)	C8—H8B	0.9700
Pr1—O11	2.629 (3)	C9—C10	1.502 (5)
Pr1—O7	2.639 (3)	C9—H9A	0.9700
Pr1—Ni2	3.5340 (6)	C9—H9B	0.9700
Ni2—N2	2.018 (2)	C10—H10A	0.9700
Ni2—O1	2.0298 (19)	C10—H10B	0.9700
Ni2—N1	2.035 (2)	C11—C12	1.459 (4)
Ni2—O3	2.0427 (19)	C11—H11A	0.9300
Ni2—O14	2.125 (2)	C12—C13	1.400 (4)
Ni2—O15	2.151 (2)	C12—C17	1.403 (4)
O1—C1	1.325 (3)	C13—C14	1.366 (5)
O2—C2	1.391 (4)	C13—H13A	0.9300
O2—C19	1.434 (4)	C14—C15	1.394 (5)
O3—C17	1.325 (3)	C14—H14A	0.9300
O4—C16	1.393 (3)	C15—C16	1.367 (4)
O4—C18	1.422 (4)	C15—H15A	0.9300
O5—N3	1.221 (4)	C16—C17	1.406 (4)
O6—N3	1.225 (4)	C18—H18A	0.9600
O7—N3	1.245 (4)	C18—H18B	0.9600
O8—N4	1.254 (4)	C18—H18C	0.9600
O9—N4	1.210 (4)	C19—H19A	0.9600
O10—N4	1.232 (4)	C19—H19B	0.9600
O11—N5	1.256 (4)	C19—H19C	0.9600
O12—N5	1.213 (4)	C20—H20A	0.9600
O13—N5	1.256 (4)	C20—H20B	0.9600
O14—C20	1.417 (4)	C20—H20C	0.9600
O14—H14O	0.8501	C21—H21A	0.9600
O15—C21	1.413 (4)	C21—H21B	0.9600
O15—H15O	0.8500	C21—H21C	0.9600
N1—C7	1.274 (4)	O16—C22	1.399 (6)
N1—C8	1.492 (4)	O16—H16O	0.8499
N2—C11	1.271 (4)	C22—H22A	0.9600
N2—C10	1.466 (4)	C22—H22B	0.9600
C1—C2	1.405 (4)	C22—H22C	0.9600
C1—C6	1.406 (4)	O17—C23	1.356 (6)
C2—C3	1.370 (4)	O17—H17O	0.8500
C3—C4	1.384 (5)	C23—H23A	0.9600
C3—H3A	0.9300	C23—H23B	0.9600
C4—C5	1.360 (5)	C23—H23C	0.9600
			
O3—Pr1—O1	67.28 (6)	O5—N3—O7	116.3 (3)
O3—Pr1—O8	138.96 (9)	O6—N3—O7	122.7 (4)
O1—Pr1—O8	146.42 (10)	O9—N4—O10	121.2 (4)
O3—Pr1—O13	91.75 (8)	O9—N4—O8	123.5 (4)
O1—Pr1—O13	76.24 (7)	O10—N4—O8	115.1 (3)
O8—Pr1—O13	114.71 (10)	O12—N5—O11	122.7 (3)
O3—Pr1—O5	76.27 (8)	O12—N5—O13	120.7 (4)
O1—Pr1—O5	94.63 (9)	O11—N5—O13	116.6 (3)
O8—Pr1—O5	77.86 (11)	O1—C1—C2	119.1 (3)
O13—Pr1—O5	167.18 (9)	O1—C1—C6	124.2 (3)
O3—Pr1—O2	129.99 (7)	C2—C1—C6	116.8 (3)
O1—Pr1—O2	62.94 (7)	C3—C2—O2	123.7 (3)
O8—Pr1—O2	89.17 (10)	C3—C2—C1	122.9 (3)
O13—Pr1—O2	72.56 (8)	O2—C2—C1	113.4 (2)
O5—Pr1—O2	111.60 (8)	C2—C3—C4	119.2 (3)
O3—Pr1—O4	63.26 (7)	C2—C3—H3A	120.4
O1—Pr1—O4	129.60 (6)	C4—C3—H3A	120.4
O8—Pr1—O4	77.13 (10)	C5—C4—C3	119.8 (3)
O13—Pr1—O4	113.66 (8)	C5—C4—H4A	120.1
O5—Pr1—O4	65.20 (9)	C3—C4—H4A	120.1
O2—Pr1—O4	166.28 (7)	C4—C5—C6	121.8 (3)
O3—Pr1—O10	143.55 (9)	C4—C5—H5A	119.1
O1—Pr1—O10	129.23 (10)	C6—C5—H5A	119.1
O8—Pr1—O10	47.88 (10)	C5—C6—C1	119.5 (3)
O13—Pr1—O10	66.87 (9)	C5—C6—C7	116.7 (3)
O5—Pr1—O10	125.74 (10)	C1—C6—C7	123.8 (3)
O2—Pr1—O10	73.27 (11)	N1—C7—C6	128.9 (3)
O4—Pr1—O10	97.47 (10)	N1—C7—H7A	115.6
O3—Pr1—O11	76.63 (8)	C6—C7—H7A	115.6
O1—Pr1—O11	111.86 (8)	N1—C8—C9	114.2 (3)
O8—Pr1—O11	97.19 (11)	N1—C8—H8A	108.7
O13—Pr1—O11	48.68 (8)	C9—C8—H8A	108.7
O5—Pr1—O11	130.31 (9)	N1—C8—H8B	108.7
O2—Pr1—O11	117.78 (8)	C9—C8—H8B	108.7
O4—Pr1—O11	65.52 (8)	H8A—C8—H8B	107.6
O10—Pr1—O11	67.06 (11)	C8—C9—C10	114.8 (3)
O3—Pr1—O7	111.93 (9)	C8—C9—H9A	108.6
O1—Pr1—O7	80.99 (10)	C10—C9—H9A	108.6
O8—Pr1—O7	69.83 (13)	C8—C9—H9B	108.6
O13—Pr1—O7	137.39 (9)	C10—C9—H9B	108.6
O5—Pr1—O7	47.29 (9)	H9A—C9—H9B	107.5
O2—Pr1—O7	65.04 (9)	N2—C10—C9	111.2 (3)
O4—Pr1—O7	108.65 (9)	N2—C10—H10A	109.4
O10—Pr1—O7	103.36 (11)	C9—C10—H10A	109.4
O11—Pr1—O7	166.94 (11)	N2—C10—H10B	109.4
O3—Pr1—Ni2	33.79 (5)	C9—C10—H10B	109.4
O1—Pr1—Ni2	33.53 (5)	H10A—C10—H10B	108.0
O8—Pr1—Ni2	163.62 (8)	N2—C11—C12	126.8 (3)
O13—Pr1—Ni2	81.66 (6)	N2—C11—H11A	116.6
O5—Pr1—Ni2	85.78 (7)	C12—C11—H11A	116.6
O2—Pr1—Ni2	96.25 (5)	C13—C12—C17	119.7 (3)
O4—Pr1—Ni2	96.77 (5)	C13—C12—C11	116.1 (3)
O10—Pr1—Ni2	148.47 (7)	C17—C12—C11	124.1 (3)
O11—Pr1—Ni2	93.93 (7)	C14—C13—C12	121.5 (3)
O7—Pr1—Ni2	98.46 (9)	C14—C13—H13A	119.3
N2—Ni2—O1	170.50 (9)	C12—C13—H13A	119.3
N2—Ni2—N1	98.11 (10)	C13—C14—C15	119.4 (3)
O1—Ni2—N1	91.11 (9)	C13—C14—H14A	120.3
N2—Ni2—O3	89.90 (9)	C15—C14—H14A	120.3
O1—Ni2—O3	80.93 (8)	C16—C15—C14	119.6 (3)
N1—Ni2—O3	171.92 (9)	C16—C15—H15A	120.2
N2—Ni2—O14	91.34 (10)	C14—C15—H15A	120.2
O1—Ni2—O14	91.21 (9)	C15—C16—O4	123.5 (3)
N1—Ni2—O14	88.46 (10)	C15—C16—C17	122.5 (3)
O3—Ni2—O14	90.33 (9)	O4—C16—C17	114.0 (2)
N2—Ni2—O15	87.12 (10)	O3—C17—C12	123.9 (3)
O1—Ni2—O15	90.18 (9)	O3—C17—C16	119.0 (2)
N1—Ni2—O15	92.60 (10)	C12—C17—C16	117.2 (3)
O3—Ni2—O15	88.82 (9)	O4—C18—H18A	109.5
O14—Ni2—O15	178.24 (9)	O4—C18—H18B	109.5
N2—Ni2—Pr1	130.30 (7)	H18A—C18—H18B	109.5
O1—Ni2—Pr1	40.58 (5)	O4—C18—H18C	109.5
N1—Ni2—Pr1	131.58 (8)	H18A—C18—H18C	109.5
O3—Ni2—Pr1	40.40 (5)	H18B—C18—H18C	109.5
O14—Ni2—Pr1	89.48 (7)	O2—C19—H19A	109.5
O15—Ni2—Pr1	90.87 (7)	O2—C19—H19B	109.5
C1—O1—Ni2	126.40 (18)	H19A—C19—H19B	109.5
C1—O1—Pr1	124.92 (17)	O2—C19—H19C	109.5
Ni2—O1—Pr1	105.89 (8)	H19A—C19—H19C	109.5
C2—O2—C19	117.8 (3)	H19B—C19—H19C	109.5
C2—O2—Pr1	118.10 (16)	O14—C20—H20A	109.5
C19—O2—Pr1	123.9 (2)	O14—C20—H20B	109.5
C17—O3—Ni2	125.58 (17)	H20A—C20—H20B	109.5
C17—O3—Pr1	124.57 (17)	O14—C20—H20C	109.5
Ni2—O3—Pr1	105.82 (8)	H20A—C20—H20C	109.5
C16—O4—C18	116.6 (2)	H20B—C20—H20C	109.5
C16—O4—Pr1	116.51 (16)	O15—C21—H21A	109.5
C18—O4—Pr1	126.5 (2)	O15—C21—H21B	109.5
N3—O5—Pr1	100.0 (2)	H21A—C21—H21B	109.5
N3—O7—Pr1	96.4 (2)	O15—C21—H21C	109.5
N4—O8—Pr1	99.7 (2)	H21A—C21—H21C	109.5
N4—O10—Pr1	97.2 (2)	H21B—C21—H21C	109.5
N5—O11—Pr1	95.5 (2)	C22—O16—H16O	108.8
N5—O13—Pr1	99.1 (2)	O16—C22—H22A	109.5
C20—O14—Ni2	124.7 (2)	O16—C22—H22B	109.5
C20—O14—H14O	99.9	H22A—C22—H22B	109.5
Ni2—O14—H14O	123.9	O16—C22—H22C	109.5
C21—O15—Ni2	126.6 (2)	H22A—C22—H22C	109.5
C21—O15—H15O	114.5	H22B—C22—H22C	109.5
Ni2—O15—H15O	110.8	C23—O17—H17O	96.4
C7—N1—C8	114.4 (3)	O17—C23—H23A	109.5
C7—N1—Ni2	123.8 (2)	O17—C23—H23B	109.5
C8—N1—Ni2	121.7 (2)	H23A—C23—H23B	109.5
C11—N2—C10	116.5 (3)	O17—C23—H23C	109.5
C11—N2—Ni2	125.3 (2)	H23A—C23—H23C	109.5
C10—N2—Ni2	117.9 (2)	H23B—C23—H23C	109.5
O5—N3—O6	121.0 (4)		

### Hydrogen-bond geometry (Å, °)

**Table:**

*D*—H···*A*	*D*—H	H···*A*	*D*···*A*	*D*—H···*A*
O14—H14O···O16	0.85	1.81	2.661 (4)	180
O15—H15O···O6^i^	0.85	2.28	3.128 (5)	180
O16—H16O···O17^ii^	0.85	1.87	2.720 (6)	179
O17—H17O···O13	0.85	2.05	2.905 (5)	180

Symmetry codes: (i) −*x*, −*y*+2, −*z*; (ii) −*x*+1, *y*+1/2, −*z*+1/2.
